# Body Site Is a More Determinant Factor than Human Population Diversity in the Healthy Skin Microbiome

**DOI:** 10.1371/journal.pone.0151990

**Published:** 2016-04-18

**Authors:** Guillermo I. Perez Perez, Zhan Gao, Roland Jourdain, Julia Ramirez, Francesca Gany, Cecile Clavaud, Julien Demaude, Lionel Breton, Martin J. Blaser

**Affiliations:** 1 Department of Medicine, New York University Langone Medical Center, New York, NY, United States of America; 2 L’Oreal Research and Innovation, Aulnay-sous-Bois, France; 3 Memorial Sloan Kettering Cancer Center, New York, NY, United States of America; 4 L’Oreal Research and Innovation, Clark, NJ, United States of America; 5 New York Harbor Department of Veterans Affairs Medical Center, New York, NY, United States of America; University of Ulster, UNITED KINGDOM

## Abstract

We studied skin microbiota present in three skin sites (forearm, axilla, scalp) in men from six ethnic groups living in New York City. **Methods.** Samples were obtained at baseline and after four days following use of neutral soap and stopping regular hygiene products, including shampoos and deodorants. DNA was extracted using the MoBio Power Lyzer kit and 16S rRNA gene sequences determined on the IIlumina MiSeq platform, using QIIME for analysis. **Results.** Our analysis confirmed skin swabbing as a useful method for sampling different areas of the skin because DNA concentrations and number of sequences obtained across subject libraries were similar. We confirmed that skin location was the main factor determining the composition of bacterial communities. Alpha diversity, expressed as number of species observed, was greater in arm than on scalp or axilla in all studied groups. We observed an unexpected increase in α-diversity on arm, with similar tendency on scalp, in the South Asian group after subjects stopped using their regular shampoos and deodorants. Significant differences at phylum and genus levels were observed between subjects of the different ethnic origins at all skin sites. **Conclusions.** We conclude that ethnicity and particular soap and shampoo practices are secondary factors compared to the ecological zone of the human body in determining cutaneous microbiota composition.

## Introduction

The skin, a unique barrier to the external environment, is the largest and most exposed organ of the human body, representing one of the most extensive of our endogenous microbial habitats [1–5]. A wide variety of microbes including viruses [6], bacteria [1–3] and fungi [7,8] are capable of colonizing the skin, with differing distributions. Variations in the cutaneous microbiota of healthy individuals are associated with the ecological zones of the skin, including the sebaceous, dry, or moist environments [3,9]. Differences in skin microbiota also have been associated with host factors, including age, diet, and gender, as well as environmental factors, such as climate and geographic location [4,10]. Skin microbiota differences have also been associated with conditions such as dandruff, acne, psoriasis, atopic dermatitis [7, 11–14]. Despite the variation in environmental factors, less temporal variability is observed in the same individual than inter-individual variability at the same skin site [1, 15].

The study of the cutaneous microbiota has been limited due to the methodologies based on culture used in the past century. Culture methods and identifying taxonomic classification through phenotypic characteristics including microscopic features and biochemical tests [16, 17], provided the basis for our knowledge of the microbiota, but was biased by differences in culturability of the taxa. Advances in DNA processing and analysis have allowed the study of bacterial communities by sequencing specific regions of the 16S ribosomal RNA with increasing depth [2, 10, 12, 18]. The sequence data can be used in analyses of phylogeny and bacterial community structure, without the bias of culture.

Investigation of healthy skin microbiota has been mostly limited to US populations with little or no indication of the ethnic background of the studied subjects [1, 15, 19, 20]. Recent studies have compared hand bacterial communities in human populations with contrasting environmental and socio-economic differences, including hand-washing frequency; differences in use of soap, deodorant, and bactericidal hand gels, among other domestic practices, may partially explain the variation observed [21, 22]. Similarly, comparison of forearm skin in US subjects and in Amerindian subjects with little or no modernization in lifestyles has shown substantial differences in bacterial populations [23, 24].

We characterized the diversity of cutaneous microbiomes in several population groups living in New York City. To avoid potential gender-based variation in skin and hair treatments, as well as differences in menstrual cycles among women, we designed a study of men of different ethnicity and country of origin. Samples were obtained from three cutaneous sites (forearm, axilla, scalp) representative of the major ecological zones -dry, moist, and sebaceous- and bacterial communities were studied by analysis of high throughput sequencing data. Here we showed that skin microbiome is primarily influenced by the ecological zone of the human body, while at each body site, host origin is a factor in determining cutaneous microbiota composition.

## Materials and Methods

### Sample collection

The experimental design of this study is described in S1 Fig. We enrolled 110 men belonging to one of 6 ethnic groups: Caucasian-American (US-borne, of European ancestry), African-American, and men born in Africa (African Continental), East Asia, Latin America, or South Asia. The study subjects were all healthy adult males, without a history of dandruff or other skin condition. From each subject, skin samples were obtained 72 hours apart from the scalp (occiput), axilla, and inner forearm (extensor surface) using a swabbing technique with duplicate swabs, as described [2], and used in the Human Microbiome Program (HMP) analyses [25]. Samples were obtained before (baseline: time 1) and after subjects stopped using their regular shampoos and deodorants when they used a generic neutral soap (follow-up; time 2). The generic neutral soap was used for the whole body and was used any time that the subject took a bath or a shower. Each swab head was placed in an Eppendorf tube and frozen at -20°C until DNA was extracted. The study was approved by the Institutional Review Board at NYU Langone Medical Center, according to the principles of the Declaration of Helsinki and participants provided their written informed consent to participate in this study.

### Sample processing

DNA was extracted from the heads of both skin swabs from the same cutaneous location in a single tube using the PowerLyzer Power Soil DNA Isolation Kit (MoBio Laboratories, Carlsbad CA), according to the manufacturer’s protocol. Samples were divided into aliquots for qPCR (for future studies) and for Illumina sequencing.

### High throughput DNA sequencing

For each DNA sample, the V4 region of bacterial 16S rRNA genes was amplified in triplicate reactions, using the universal bacterial primer set 515F/806R, which nearly universally amplifies bacterial and archaeal 16S genes [26–28]. Amplicons from each sample were quantified using a PicoGreen dsDNA Assay Kit (Invitrogen, Eugene OR). Equal amounts of DNA from each sample were pooled, followed by PCR purification (Qiagen, Hilden, Germany). DNA concentrations in these pools were quantified with the Qubit high sensitivity dsDNA Assay (Invitrogen), and then combined at equal concentration. DNA sequencing was performed on an Illumina MiSeq instrument located in the NYULMC Genome Technology Center.

### Data analysis

Sequence data were processed with QIIME v1.7.0 [29] as described [30]. Briefly, sequences were demultiplexed and quality filtered using default QIIME parameters, and clustered into operational taxonomic units (OTUs) with a sequence similarity threshold of 97% with UCLUST [31] within QIIME. The sequence reads were clustered against the May 2013 release of the Greengenes [32, 33] 97% reference data set (http://greengenes.secondgenome.com). Linear discriminant analysis effect size (LEfSe) [34] was used, via the Galaxy Browser, to detect significant changes in relative abundance of the microbial taxa among different groups. To reduce the number of features, the analysis was limited to taxa with relative abundance ≥ 0.1% in any sample. Significance thresholds were performed at the default settings.

### Statistical Analysis

Significance was determined using one-way Anova for multiple comparisons, Mann-Whitney t-test, Friedman test, and Chi-Square test, as appropriate; differences p < 0.05 were noted as significant. All data analysis was performed using Graph Pad Prism software.

## Results

### Demographic characteristics of the studied groups and data sequence analysis

We enrolled a total of 110 men from six ethnic groups (11 to 25 individuals per group). The studied volunteers were either native to the US or newcomers, who spent 4–11 years in the US, with a mean age of 28.7 years (range 21–50) (S1 Table and S1 Fig). The groups ranged in mean age from 24.0 (East Asia) to 37.9 (South Asia). Overall, the East Asians were significantly younger than the Latin American and South Asian subjects (p <0.05 for both). The South Asian men were significantly older than all of the other studied groups, except for the African-Continental group. Among the groups of subjects born outside of the US, the East Asian men had most recently arrived, with a mean of 4.7 years in the US.

Of the 11,115,309 Illumina reads from the V4 region of bacterial 16S rRNA genes that passed the QIIME quality filters, 98.5% (10,952,313) matched a reference sequence at ≥97% nucleotide sequence identity, and those failing to match within this threshold were discarded. Taxonomy was assigned to the retained clusters of operational taxonomic units (OTUs) based on the Greengenes reference sequence, and the Greengenes tree was used for all downstream phylogenetic community comparisons. For 15 (2.3%) of the 660 samples studied, a small number of sequences were obtained (<3200), and they were not included in the analysis. In the remaining 645 samples the total number of sequences per subject per site from each ethnic group ranged from 3,204 to 87,590, with a mean of 16,980 and a median of 15,519 sequences per sample. To avoid biases caused by differences in sequencing depth of samples, analyses were conducted on data rarefied to 3,200 sequences/sample (S2 Fig).

### Analysis of skin microbiome variation by skin location

**Fig 1:**
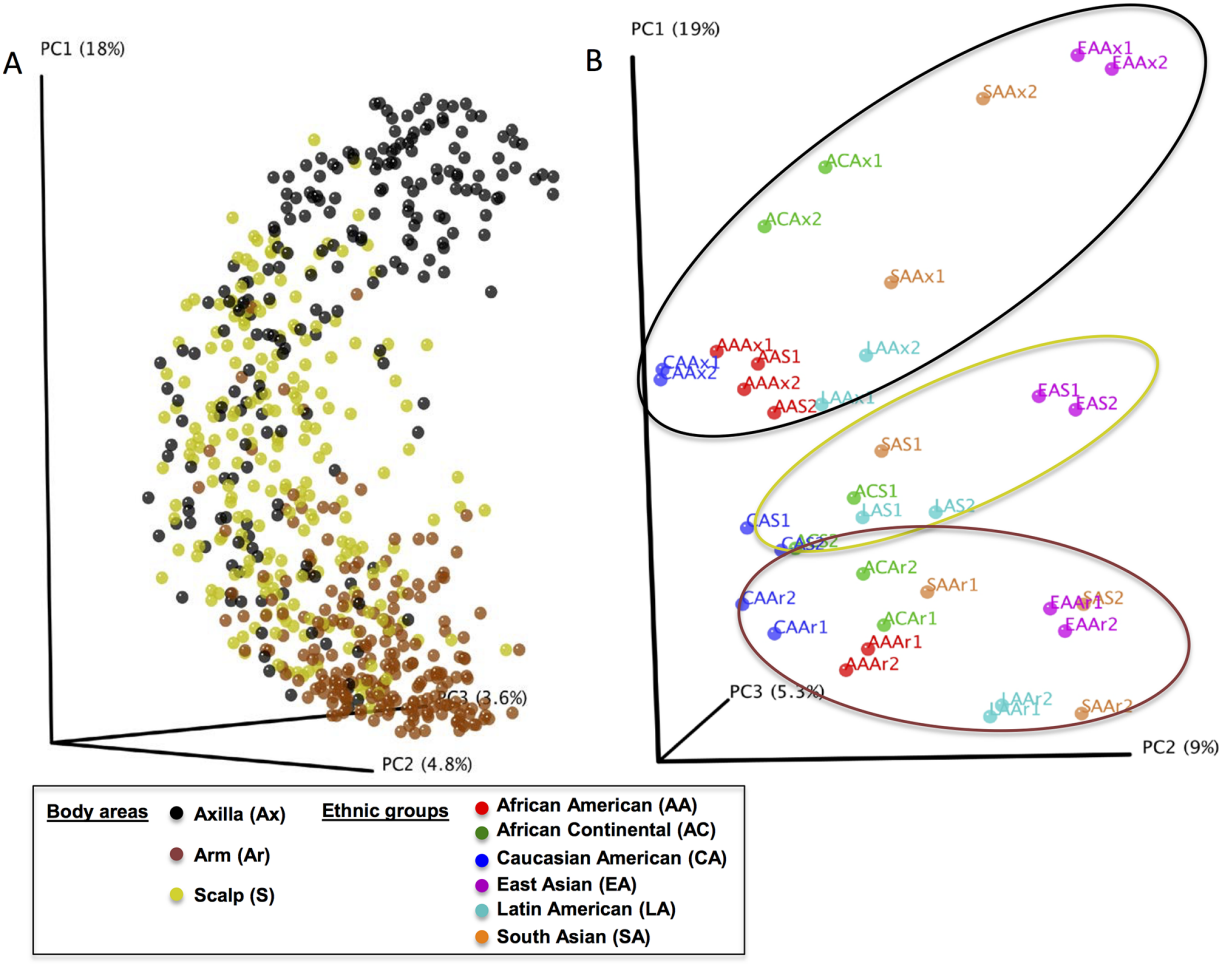
Cutaneous microbial diversity in 645 samples from 110 subjects, based on skin location and beta diversity analysis over time, by ethnic group. The sequence depth is 3200/sample. **Panel A.** PCoA of clustering by sample location, based on unweighted pairwise UniFrac distances. The skin locations were Scalp [S (n = 215 samples)]; Arm [Ar (n = 215)]; Axilla [Ax (n = 215)]. **Panel B**. Analysis by ethnic group, including African-American [AA (n = 18 subjects/site)], East Asian [EA (n = 25)], Caucasian-American [CA (n = 16)], Latin American [LA (n = 16)], African-Continental [AC (n = 11)], and South Asian [SA (n = 19)]. PCoA of clustering of skin samples combining the three cutaneous sites, and the two time points; 1 = baseline and 2 = follow-up. Ellipses generally describe: axilla (black), scalp (yellow), and Arm (brown). **Key to explain sites sampled and ethnic groups in all figures:** The skin locations are colored as follows: yellow: Scalp (S); Brown: Arm (Ar); Black: Axilla (Ax). Ethnic groups are colored as: Orange: South Asian (SA); Light blue: Latin-American (LA); Purple: East Asian (EA); Red: African-American (AA); Blue: Caucasian-American (CA); Green: African-Continental (AC).

Once the sequences were processed and OTUs were assigned, we analyzed the clustering of the 645 skin samples based on unweighted Unifrac distances, and visualized by Principal Coordinates Analysis (PCoA). As expected, the analysis shows that skin samples clustered according to skin location (Fig 1, **Panel A**).

Phylogenetic distance was used to measure the alpha-diversity in the 645 samples according to each of the three locations. The arm samples showed the highest diversity, followed by the scalp; the axilla samples showed the lowest diversity (**Panel A in** S3 Fig); the three sites differed significantly (p<0.05). In addition, significant differences were observed between the skin locations for each population group, except that differences between scalp and axilla in African-Americans were not significant (data not shown). Because of the clear differences in clustering based on skin location, we performed the remainder of the analyses of the ethnic group-specificity for each skin location separately, and a second analysis was based on time of collection, before (Time 1) and after three to four days of neutral soap use and stopping regular hygiene products (Time 2).

### Assessment of beta diversity in the six study groups for the three skin sites and in relation to repeat sampling

We examined the community structure (beta diversity) of the skin samples using unweighted UniFrac distances according to body location, and across the two times of sampling, for each group. For this analysis, we included all the sequencing results for each site and for both times for all subjects within a specific group (Fig 1, **Panel B**). Several features were observed: the samples primarily clustered by skin site, and in most cases, for each group of subjects, the samples collected at the two times points were highly similar. One interesting observation is that both the scalp and axilla skin microbiomes of the African-American group were clustered closely to each other. In addition, the scalp microbiome at time 2 in the South Asian group was significantly different (p = 0.01; Adonis test) from time 1 and closer to the arm microbiome at times 1 and 2. In analyses of the community variation using pairwise unweighted UniFrac distances among skin locations (**Panel B in** S3 Fig), the arms had significantly less intragroup variation than the axilla or the scalp, and the axilla had the greatest intragroup variation. In terms of intergroup variation, the mean pairwise distances were significantly greater for arm vs axilla than for the intragroup differences, providing evidence that the community structures of these two sites are distinct.

### Analysis of diversity of the cutaneous microbiome over time

**Fig 2:**
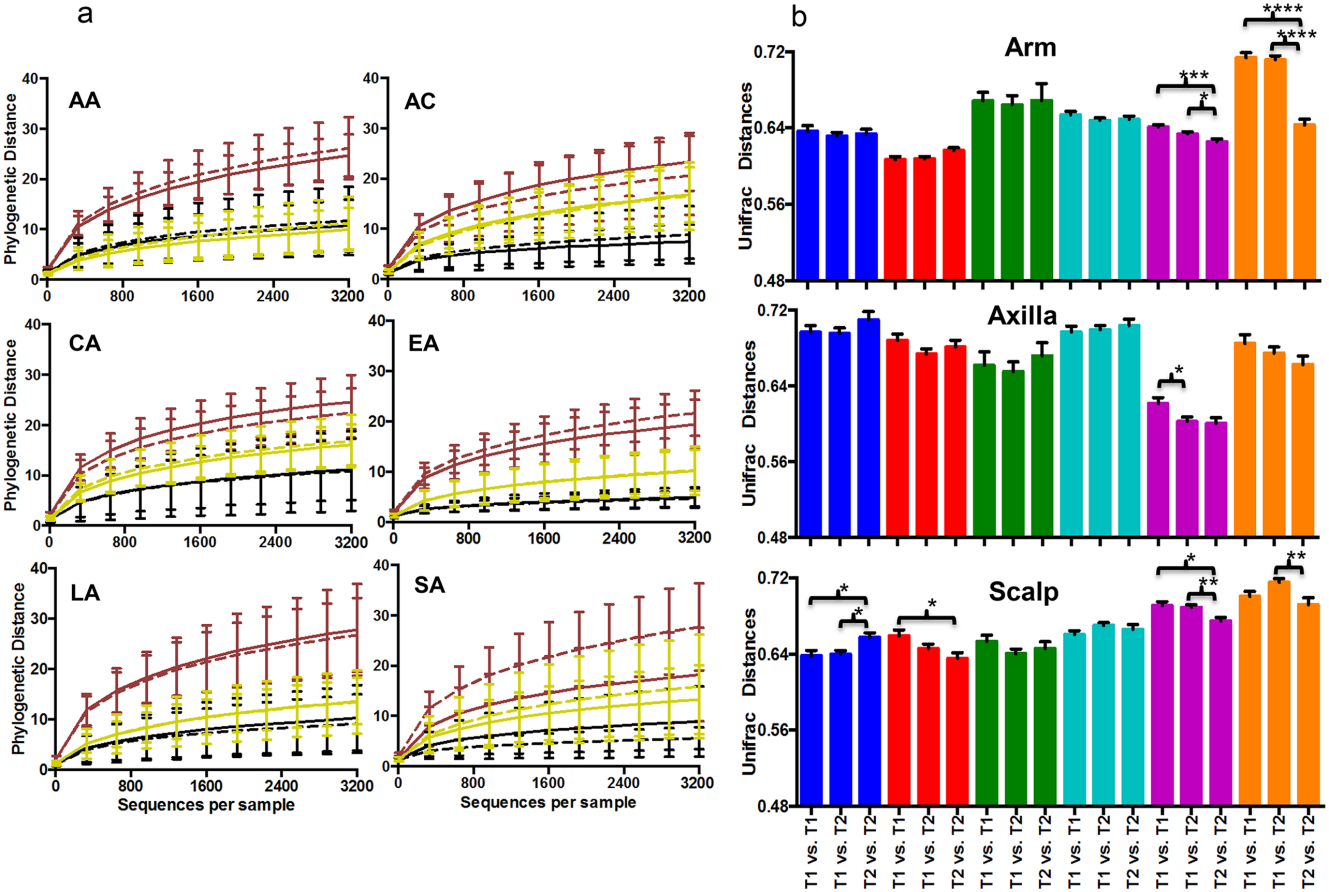
Comparison of diversity of the cutaneous microbiota over time, by ethnic group. **Panel A.** Rarefaction analysis of cutaneous microbiota in relation to sampling time in samples from arms (Brown), axilla (Black) and scalp (Yellow), African-American (AA, n = 108 samples); African-Continental (AC, n = 66); Caucasian-American (CA, n = 96); East-Asian (EA, n = 150); Latin-American (LA, n = 114); South-Asian (SA, n = 96). Rarefaction analysis represented by phylogenetic distance. The solid and dashed lines indicate samples collected before (T1) and after (T2) a special soap wash was used, respectively. Only a significant difference between time-points was found for the SA group. **Panel B.** Intra- and inter-group beta-diversity over time. Mean (±SD) pairwise unweighted UniFrac distances are shown. The ethnic groups are colored as described in Fig 1. Significance was determined by one-way ANOVA with the Tukey method for correction for multiple comparisons (*<0.05; **<0.01; ***<0.001; ****<0.0001).

As determined by analysis of phylogenetic distances, we did not find any significant difference in alpha diversity within each of the ethnic groups at each skin site, comparing the two time points, with one exception (Fig 2, **Panel A**); a significant difference in number of observed species was found between the arm samples before (time 1) and after stopping the use of shampoos and deodorants (time 2) in the South Asian group (p = 0.03). In contrast, alpha diversity expressed as the number of observed species, Chao index, or phylogenetic distance amongst the South Asian group was similar at the three skin sites. For the other 5 ethnic groups studied, using all of the different metrics to assess alpha diversity, the three skin sites at time 1 all significantly differed from each other (data not shown).

Because of concerns about age differences, we performed a sub-analysis of beta-diversity differences related to age at each of the three skin sites (S4 Fig). There were no significant differences between the inter-group Unifrac distances. We only observed consistently significant differences in the intra-group distances for the three sites between group 1 (<25 years old; n = 77), group 2 (25 to 29 years old; n = 59) and group 3 (>30; n = 79). Next, changes in bacterial community structure over time were also examined. In general (in 43/54 comparisons), the mean pairwise UniFrac distances were not significantly different for the 2 time points at each skin location (Fig 2, **Panel B**). However, there were significant (p value <0.001) differences between the 2 time-points in samples from the arm in the East Asia and South Asia groups, and in scalp in the African-Continental, African-American and the East Asia groups.

Next, we performed a similar analysis of the community variation between time 1 and time 2 based on UniFrac distances (**Panel C in** S3 Fig) in the same individuals (homologous) and between all the subjects (heterologous). As expected, for all three skin sites, compositional differences were significantly greater for different individuals than the same persons at the two time points. This analysis provides further confirmation of the relative identity and stability of the cutaneous microbiome in each individual at each of the three sites examined, with a neutral and uniform cleansing regimen.

### Analysis of the diversity of the cutaneous microbiome by ethnic group

**Fig 3:**
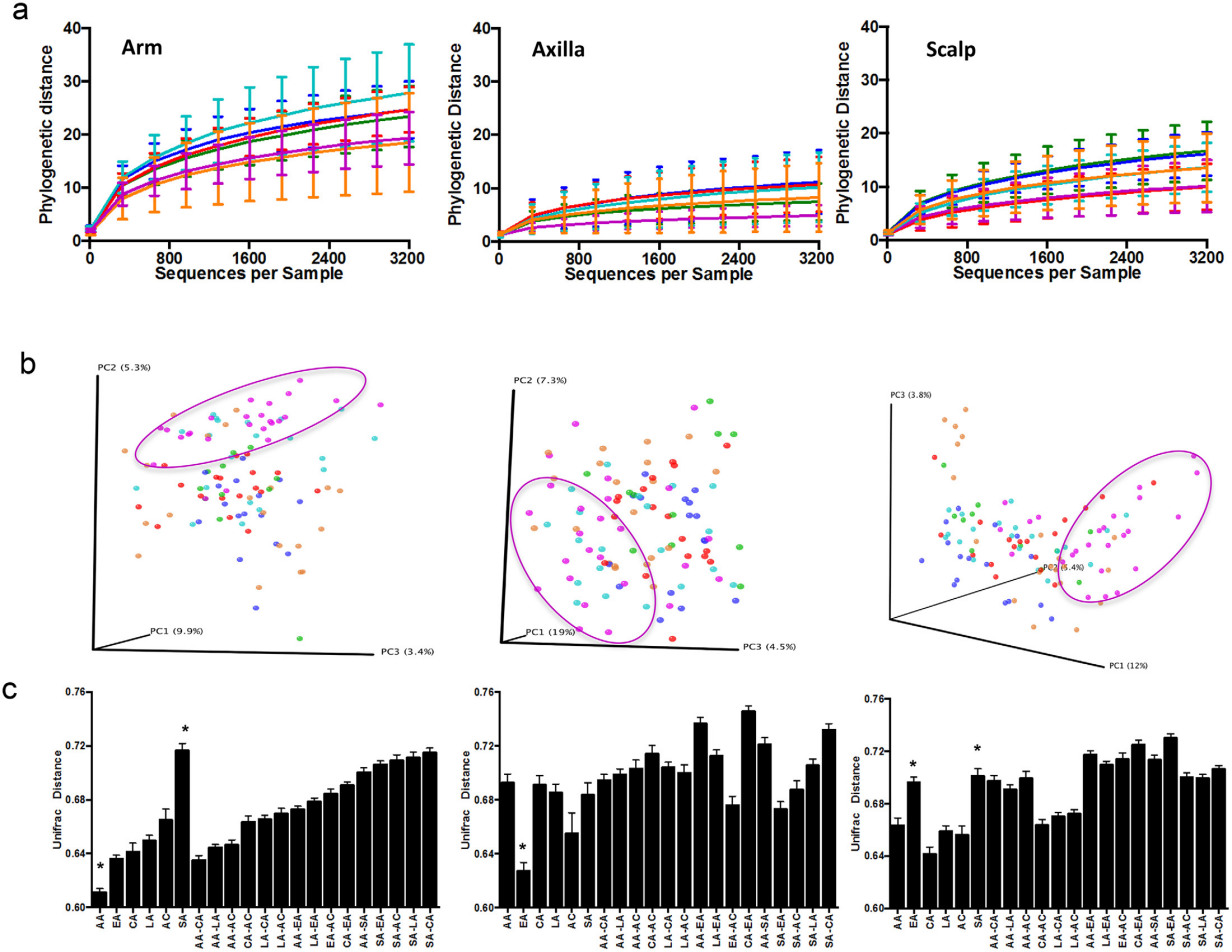
Analysis of cutaneous microbiota by ethnicity. The results are from 6 ethnic groups, including Caucasian-American (n = 20/site), African-American (n = 18/site), American-Continental (n = 11/site), Latin American (n = 16/site) East Asian (n = 25/site), and South Asian (n = 20/site). **Panel A.** Alpha diversity represented by phylogenetic distances. Arm, significant differences were found between EA vs. LA and AA; and SA vs. LA. Axilla, between EA vs. AA, LA and CA. Scalp, between EA vs. AC and CA; AA vs. AC and CA. The ethnic groups are colored as described in Fig 1. **Panel B.** PCoA of clustering by ethnic group, based on unweighted pairwise Unifrac distances. The circles represent the distribution of the East Asian samples. **Panel C.** Intra- and inter-group beta-diversity analysis on skin location at baseline. Mean (±SD) pairwise unweighted UniFrac distances shown. The * symbol indicate that the intra-group beta diversity was significantly different (p<0.001).

To assess baseline differences between ethnic groups, including the use of deodorants and shampoos, analyses among the different groups were performed using the samples obtained at Time 1 (Fig 3). Similar to the observations above (S3 Fig), alpha-diversity measured by Phylogenetic Differences (PD) was higher in the arm than in scalp or axilla, independent of the group (Fig 3, **Panel A**). The lowest alpha diversity was observed in the axilla for all 6 groups. PD of the samples from the three skin sites in the East Asian men was significantly lower than for the other groups, including African-American and Latin-American in arm; African-American, Caucasian-American and Latin-American in axilla, and African-Continental and Caucasian-American in scalp (p<0.05). Significant differences in PD between the six groups included South-Asians significantly lower than Latin-Americans in arm; and African-Americans significantly lower than African-Continental and Caucasian-Americans in scalp (Fig 3, **Panel A**). Studies based on numbers of observed species as another metric to ascertain alpha diversity showed similar results (data not shown).

Next, we examined community structure of the cutaneous samples using unweighted Unifrac distances according to both body location and the origin of the study subjects (Fig 3, **Panel B and C**). Using PCoA visualization (Fig 3, **Panel B**), no clear patterns of clustering were found among the six groups. However, the samples from the East Asian men, the group that arrived the most recently in the US, were less distant from each other than from samples in the other groups. Comparing the intra- and inter-group differences (Fig 3, **Panel C**), for the axilla, East Asian samples were most homogeneous, and the other groups were more similar to each other. For the arm samples, African-Americans showed the greatest homogeneity while South Asians showed the least. For the scalp, the heterogeneity of the samples from South Asian and East Asian men were significantly greater than in the other four groups. Among the groups, South Asian samples showed the greatest heterogeneity at two (arm and scalp) of the three sites, and major differences compared to the samples from the other groups (Fig 3, **Panel C**). These data showed little relationship between the homogeneity or heterogeneity of the microbiome and date of arrival in the US. Thus, we conclude that at each body site, skin microbiome is primarily influenced by the host origins.

### Taxonomic changes in the cutaneous microbiota and comparative analysis

**Fig 4:**
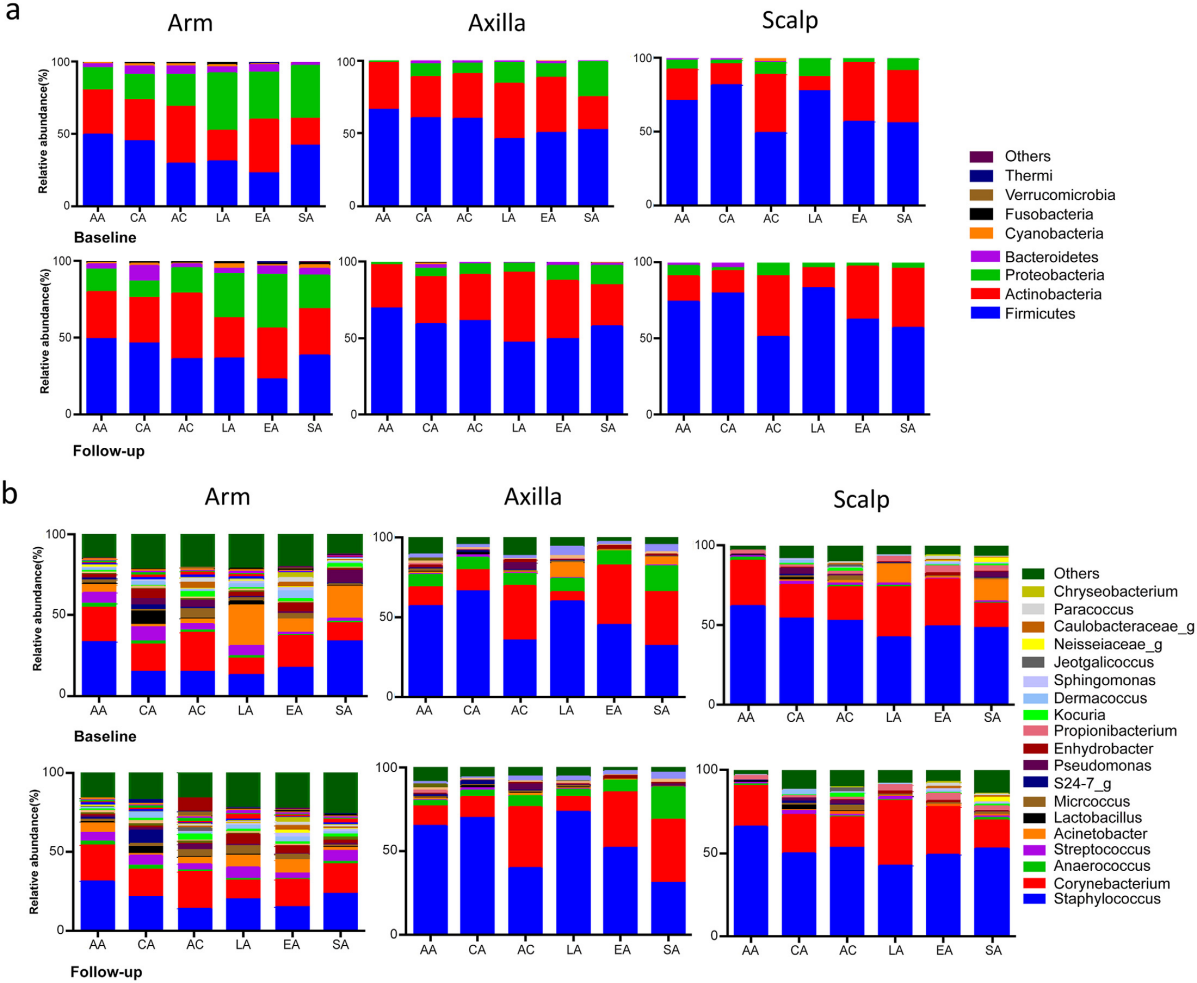
Taxonomic analysis of cutaneous microbiota from 110 subjects in six different subject groups. **Panel A.** By site (arm, axilla, scalp) at the phylum level. A total of 41 phyla were found. The sequences for the dominant 9 phyla (>0.1% in any group) accounted for >99.7% of total sequences in all ethnic groups. Baseline: Samples collected before special soap wash used. Follow-up: Samples collected after special soap wash used. **Panel B:** Genus level. A total 726 genera were detected; only predominant genera (Mean>0.01%) are shown.

For all three skin locations and times, Firmicutes and Actinobacteria together were the dominant phyla, followed by Proteobacteria. These three phyla represent >90% of all the resident taxa. Taxonomic analysis of the samples at baseline and at follow-up showed substantial stability of the major phyla at each specific skin site for each ethnic group (Fig 4, **Panel A**). Only the South Asian men had substantial variation between the baseline and follow-up samples, as observed in all three locations (Fig 4, **Panel A**). When we compared the number of genera present in each skin site across all of the groups, the axilla had the fewest (726), the arm had the most (1284), and the scalp was intermediate (1034). These differences in the number of genera were consistent at both time points (Fig 4, **Panel B**). Inter-site differences in the most abundant genera (present in >0.1%) among the six groups of volunteers included *Staphylococcus* and *Corynebacterium* with the most substantial variation, with > 80% on the axilla and the scalp, but < 50% on the arm. Genus *Propionibacterium* was more abundant in the axilla and scalp than on the arm, except in African-Continental and Latin American men. Most of the study groups had stable skin microbiota, independent of their country of origin, at the genus level, across the times studied with only the single exception discussed above. Particularly on the arm and scalp of the South Asian men, the relative abundance of genus *Acinetobacter* was decreased, with several replacing genera (Fig 4, **Panel B**); this confirmed the major differences observed at the global level, based on the UniFrac distances.

**Fig 5:**
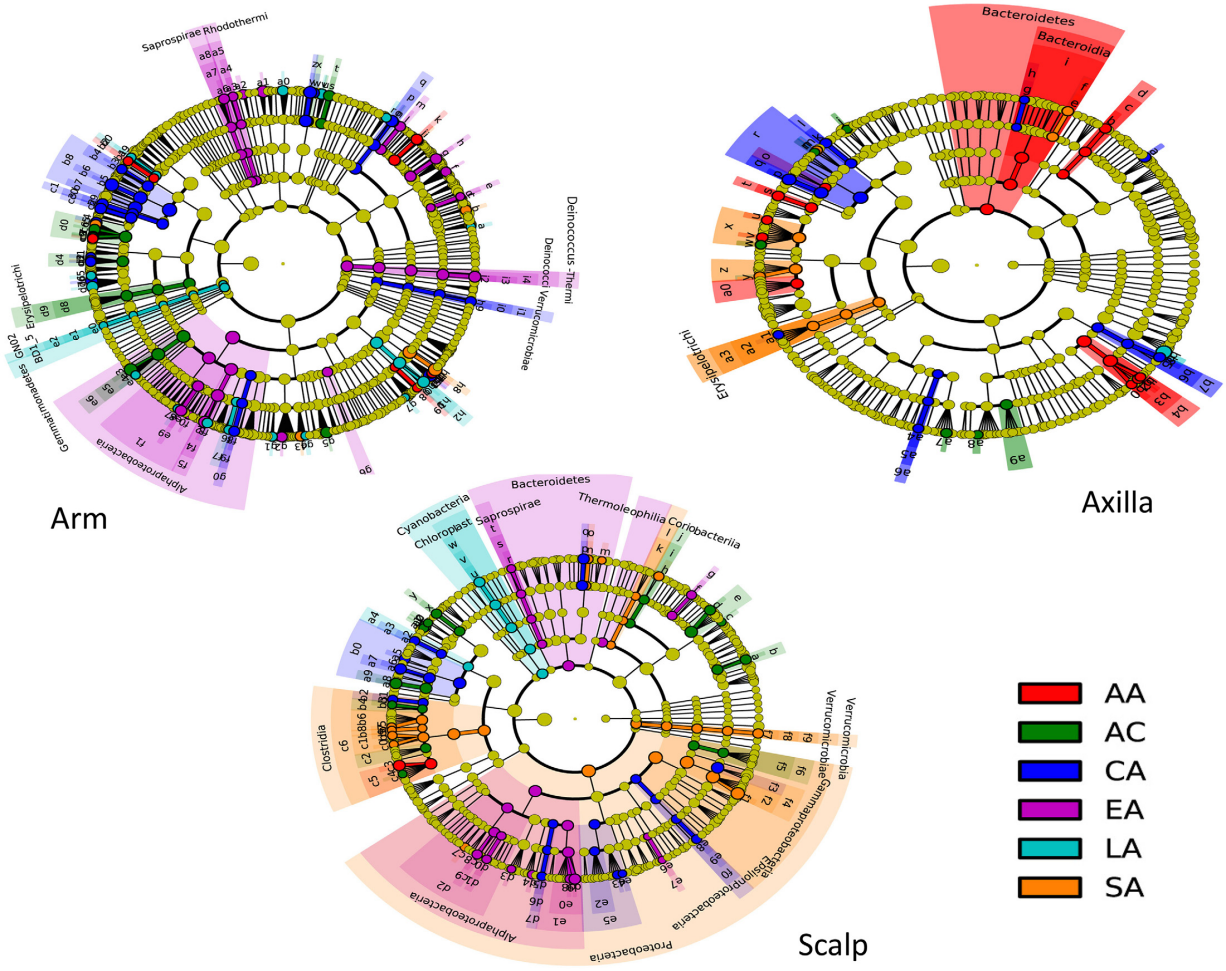
Comparison of microbiota differences by ethnic group, analyzed by LEfSe using baseline samples. Taxa with relative abundance ≥ 0.1% present in at least one sample in each location were included. The cladograms indicate the phylogenetic distribution of the microbial lineages associated with ethnic group, with lineages with Linear Discriminant Analysis (LDA) score ≥2.0 displayed. Significance differences for each ethnic group of the most abundant class are indicated by color, as indicated in the key. Each node’s diameter is proportional to the taxon’s abundance. The strategy of multiclass analysis is non-strict (≥ one class differential). Nodes represent phylogenetic levels from domain to genus (from inside out) (AA, n = 18 subjects; AC, n = 11; CA, n = 16; EA, n = 25; LA, n = 19; SA, n = 20).

We performed comparative analyses of the relative taxa abundances in the samples at baseline across the groups studied (Fig 5). There were significant differences in the relative taxa abundances associated with both skin location and ethnic group. The largest differences in relative taxa abundances between the ethnic groups involved the scalp; the South Asian men showed the most divergent taxa compared to the other groups, but multiple differences involving diverse taxa were significant across other groups. The arm showed other major differences, with the fewest in the axilla. The East Asian men had numerous taxa that were significantly more abundant in the arm than in the other five study groups (including Class *Alphaproteobacteria*, as well as genus *Deinococcus* from phylum Deinococci-Thermi at all 5 taxonomic levels). For the axilla samples, the most significant differences involved the African-American and Caucasian-American subjects. Thus, the ethnic groups showed a number of substantial group-specific taxonomic differences.

## Discussion

Compared to other body sites, such as in the gastro-intestinal (GI) tract, for example, the biomass of microbial DNA harvested from cutaneous sites is relatively low [35], and techniques for sampling have ranged from skin biopsies to swabbing [15, 20, 36]. However, in this study, the extent and quality of the bacterial DNA extraction, as evaluated by the mean number of sequences obtained, did not differ for the three cutaneous sites in the six different groups studied, and the range of taxa identified at substantial levels (7/42 phyla at > 0.1% representation, and 213/1362 genera at >0.01% representation) provides one form of evidence for sufficient sampling. In the absence of a gold standard to ascertain the taxa truly present at any site in humans, these observations extend the validation of the methods used for sampling, DNA extraction, and sequencing analysis. That the representation of *Propionibacterium* species in scalp colonization was lower than in prior studies [1, 37] may be partially explained by differences in 16S rRNA primers and sequencing techniques.

This study confirms prior reports [1, 15, 19, 20, 38, 39] that the major factor determining cutaneous microbiota composition is the ecological zone, and we now show that the effects of ethnicity and of particular soap and shampoo practices are secondary to ecological zone. We did not study fungal populations of the skin, since recent studies indicate domination by *Malassezia* at all sites, except for the feet (not sampled in this study) [8], and in the scalp of dandruff-affected subjects [40].

We observed that in general, the skin microbiome in each of the ethnic groups resemble the others in relation to skin site (Fig 1, **Panel B**). However, despite the overall conservation, there were clear differences among the ethnic groups, and in the group characteristics. Such differences may be explained by endogenous (immune status, sweat and sebum compositions) and/or exogenous factors (food and hygiene preferences) other than ethnicity [41, 42].

In the further analyses by ethnicity (Fig 3), the arm microbiota in African-American men was relatively homogenous in all the subjects and significantly different from all the other groups, including the African-continental group (Fig 3, **Panel C**). Similarly, the axillary microbiota of the East Asian men also was highly homogenous and significantly different from the other groups, and comparable differences were found for the South Asian subjects compared with the other ethnic groups. One explanation for such differences could be the ethnic diversity in an ABCC11 allele SNP, with high (70–95%) frequency of the A/A genotype among East Asians compared to Africans & African Americans (<5.0%) or Caucasian-American individuals (20%) [43]. ABCC11, which encodes an apical efflux pump, is critical for development of characteristic axillary odor; SNP G538A, prominent among Asians, leads to a nearly complete loss of the typical odor components in axillary sweat [44]. Other axillary microbiota differences, such as significantly lower abundance of *Staphylococcus* species and greater abundance of *Corynebacterium* species, were recently reported in subjects with the A/A genotype [45]. In any event, the amount of inter-individual variation between subjects suggest that much larger groups would need to be tested to reliably ascertain substantial differences in the cutaneous colonization patterns.

Stability of the microbiome was observed at baseline and at follow-up in most of the six population groups, results indicating that microbial communities in the skin sites sampled had minor (short-term) temporal variability among individuals. The skin microbiota was thus stable despite that subjects stopped the use of regular shampoos and deodorants for a 72-hour period. However, these results differ from a recent report [46], in which there was significant microbiota temporal variability in cutaneous sites. Potential explanations for the differences include that in the prior study, samples were collected by the study subjects themselves while in our study all samples were collected by the same researcher (JR). Second, although Flores et al. studied the variability in skin microbiota during a 10-week period, we assessed variability within three days. However, stability of the skin microbiota in the longer term has been shown [1]. Third, the sampling locations in the prior study were forehead and palms versus scalp, arm, and axilla in our study. The only temporal variability we observed involved the arm and scalp microbiome for the South Asian men. This variability may reflect cessation of the regular shampoos and soaps and the use of generic standard soap that we provided, since we did not find any other difference among the six different groups in relation to the number of showers taken daily or for any other cleansing practice.

There were few significant differences in richness, community structure, and in taxonomic representation in most of the study groups before and after they stopped using their regular shampoos and deodorants. Such observations suggest that changing soaps and shampoos may not have substantial impact on the underlying microbial population structure. However, the major differences in the South Asian group in samples before and after cessation, especially arm and scalp, indicate that such effects may occur. Determining the basis for these differences may provide insights into the plasticity of the cutaneous microbiome, and how it may be altered by specific interventions.

In conclusion, we showed that ethnicity and the use of particular soap and shampoo practices are secondary factors compared to the ecological zone of the human body in determining cutaneous microbiota composition. Whether or not the community richness differences observed across human populations of various origins have biological or clinical significance remains to be determined.

## Supporting Information

S1 Fig Study design. Description of the six population groups sampled, the study design, and the total number of samples collected.
(TIFF)

S2 Fig Depth of sampling for 645 samples from 110 study subjects. Total number of edited sequences: 10,952,313. The number of sequences per sample are: Mean ± SD: 16,980 ± 9168 (minimum: 3,204; maximum: 87,590; Median: 15,519).
(TIFF)

S3 Fig Assessment of alpha and beta diversity of the cutaneous microbiome by skin location. **Panel A:** Alpha diversity, represented by phylogenetic distance of arm (brown); scalp (yellow); and axilla (black); For 3200 sequences per sample, all differences between the three cutaneous locations are significant (p<0.05, using Student’s t-test with 1000 Monte Carlo simulations). **Panel B,** Intra- and Inter-group beta diversity of the cutaneous locations, assessed by pairwise unweighted UniFrac distances (**p value<0.01, ***p value <0.001, ****p value <0.0001). **Panel C,** Comparative analysis of beta diversity of the two time points, assessed by pairwise unweighted UniFrac distances; in the same subjects [homologous (Black)] and across different subjects [heterologous (gray)]. ****p value<0.00001.
(TIFF)

S4 Fig Effect of subject age on intergroup variation in community structure. **Panel A**. Subjects were divided into three age strata [group 1:<25y, n = 77 (lime); group 2: 25–29y, n = 59 (pink); and group 3:>30y, n = 79 (gray)] and unweighted UniFrac analyses were done for each site sampled, and results visualized by PCoA. **Panel B**. Mean pairwise unweighted UniFrac comparing intra- and inter-group differences between groups 1, 2, and 3. ***p value <0.001.
(TIFF)

S1 Table Country of origin, age and years in the US of subjects in the six study groups. ^a^ From Ecuador, n = 15 subjects and from Mexico, n = 5. ^b^ NA, not applicable because born in the US. *Significantly older than African-American, Caucasian-American, and EastAsian, p<0.02. **Significantly younger than Latin American and South Asian, p<0.04. ^Significant longer residency in the US than East Asian, and African-Continental, p<0.05.
(TIFF)
