# Supplementary figures and images for: Body Site Is a More Determinant Factor than Human Population Diversity in the Healthy Skin Microbiome
Source: PLoS One. 2016 Apr 18;11(4):e0151990. doi: 10.1371/journal.pone.0151990 (PMC4835103; doi:10.1371/journal.pone.0151990)

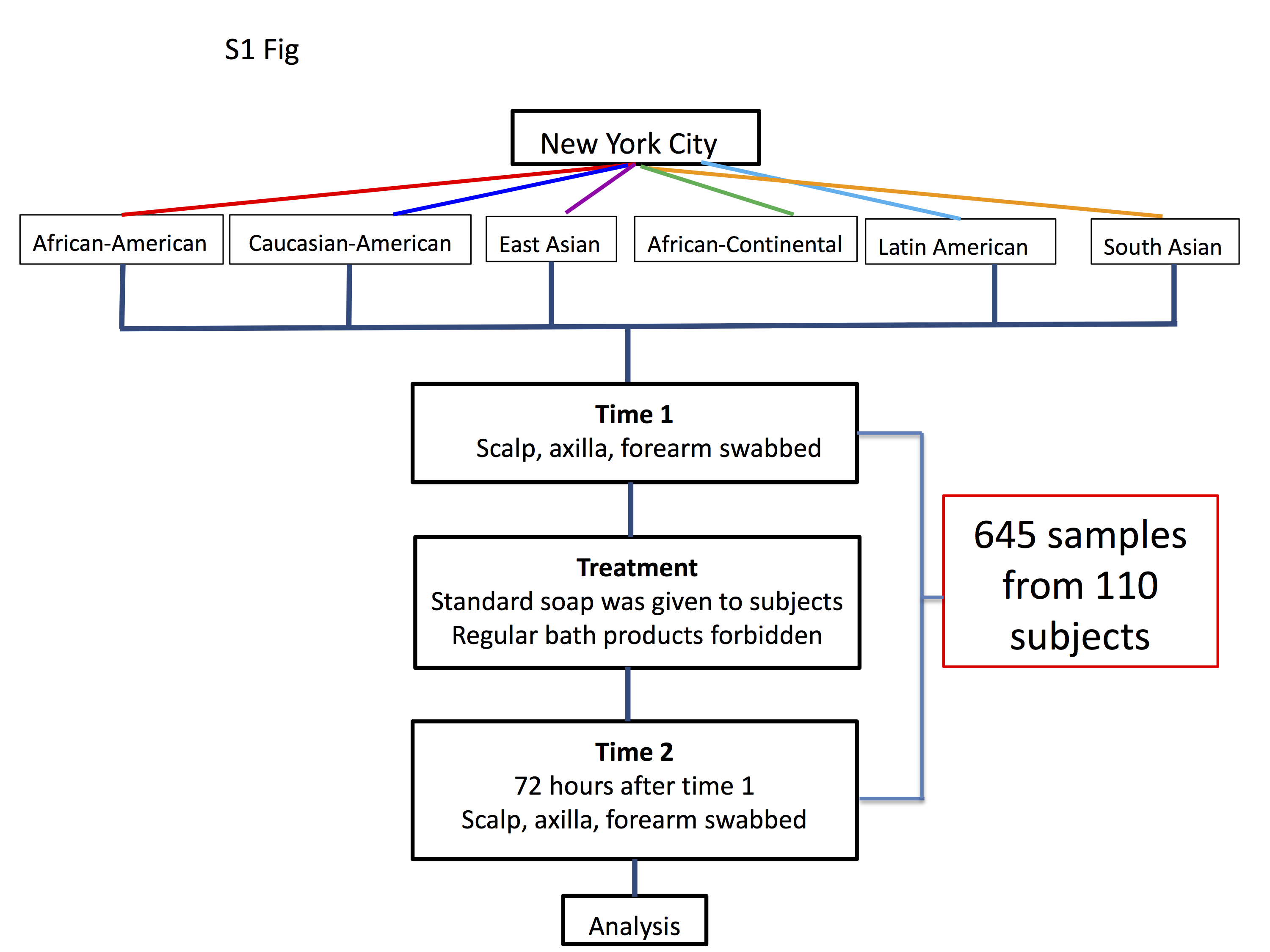

Supplement: S1 Fig — Description of the six population groups sampled, the study design, and the total number of samples collected. (TIFF) [file pone.0151990.s001.tiff]

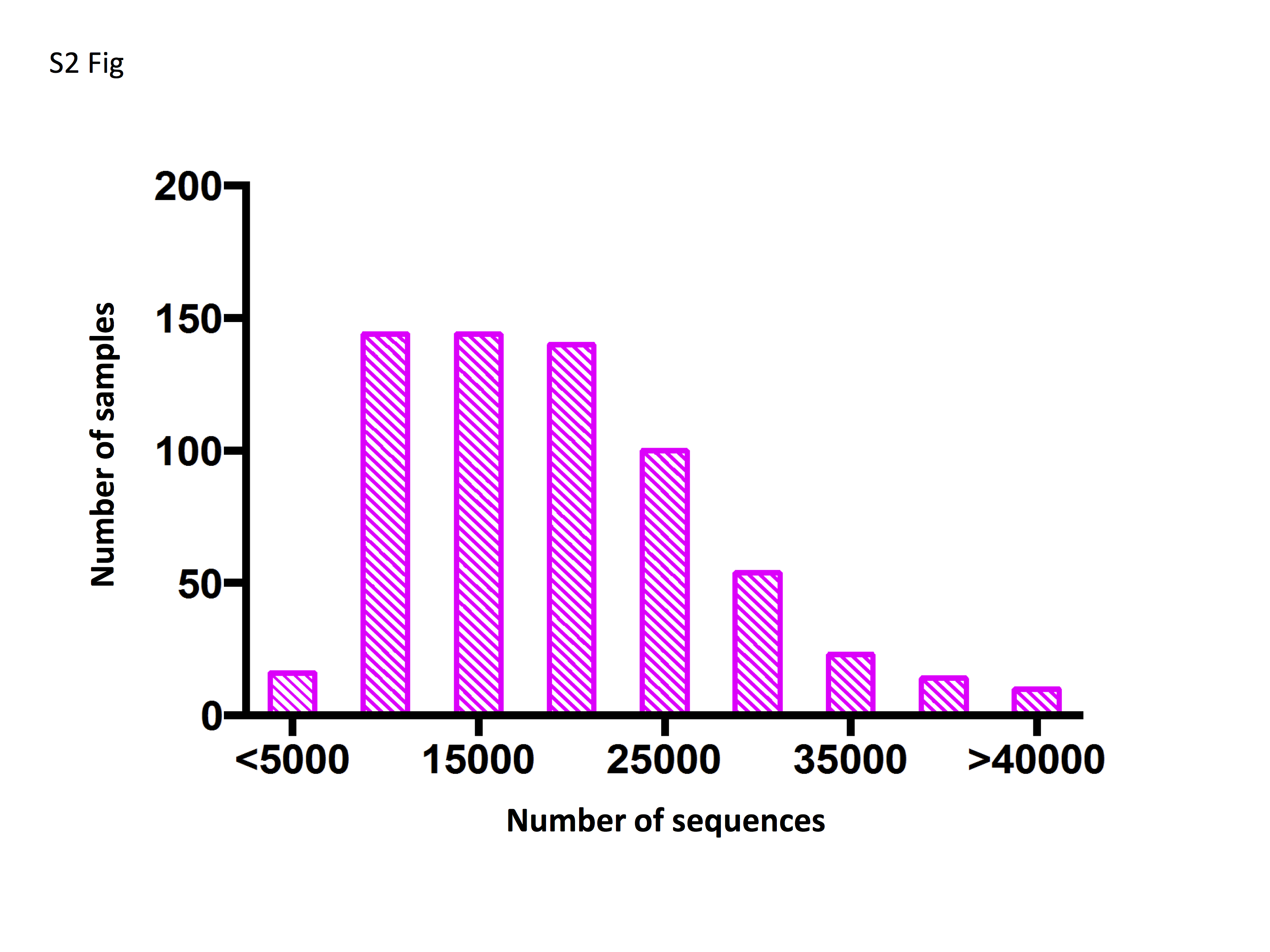

Supplement: S2 Fig — Total number of edited sequences: 10,952,313. The number of sequences per sample are: Mean ± SD: 16,980 ± 9168 (minimum: 3,204; maximum: 87,590; Median: 15,519). (TIFF) [file pone.0151990.s002.tiff]

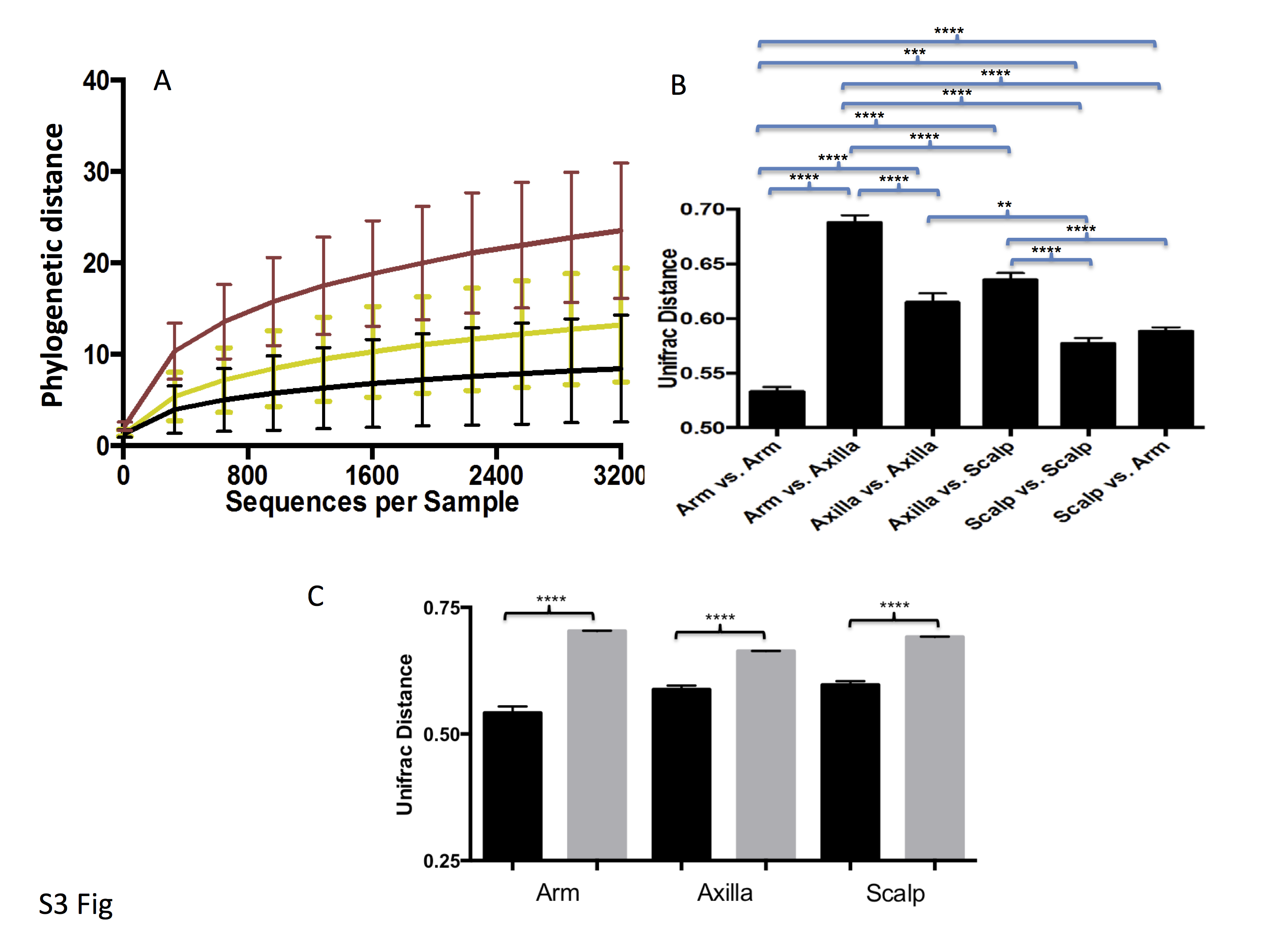

Supplement: S3 Fig — Panel A: Alpha diversity, represented by phylogenetic distance of arm (brown); scalp (yellow); and axilla (black); For 3200 sequences per sample, all differences between the three cutaneous locations are significant (p<0.05, using Student’s t-test with 1000 Monte Carlo simulations). Panel B, Intra- and Inter-group beta diversity of the cutaneous locations, assessed by pairwise unweighted UniFrac distances (**p value<0.01, ***p value <0.001, ****p value <0.0001). Panel C, Comparative analysis of beta diversity of the two time points, assessed by pairwise unweighted UniFrac distances; in the same subjects [homologous (Black)] and across different subjects [heterologous (gray)]. ****p value<0.00001. (TIFF) [file pone.0151990.s003.tiff]

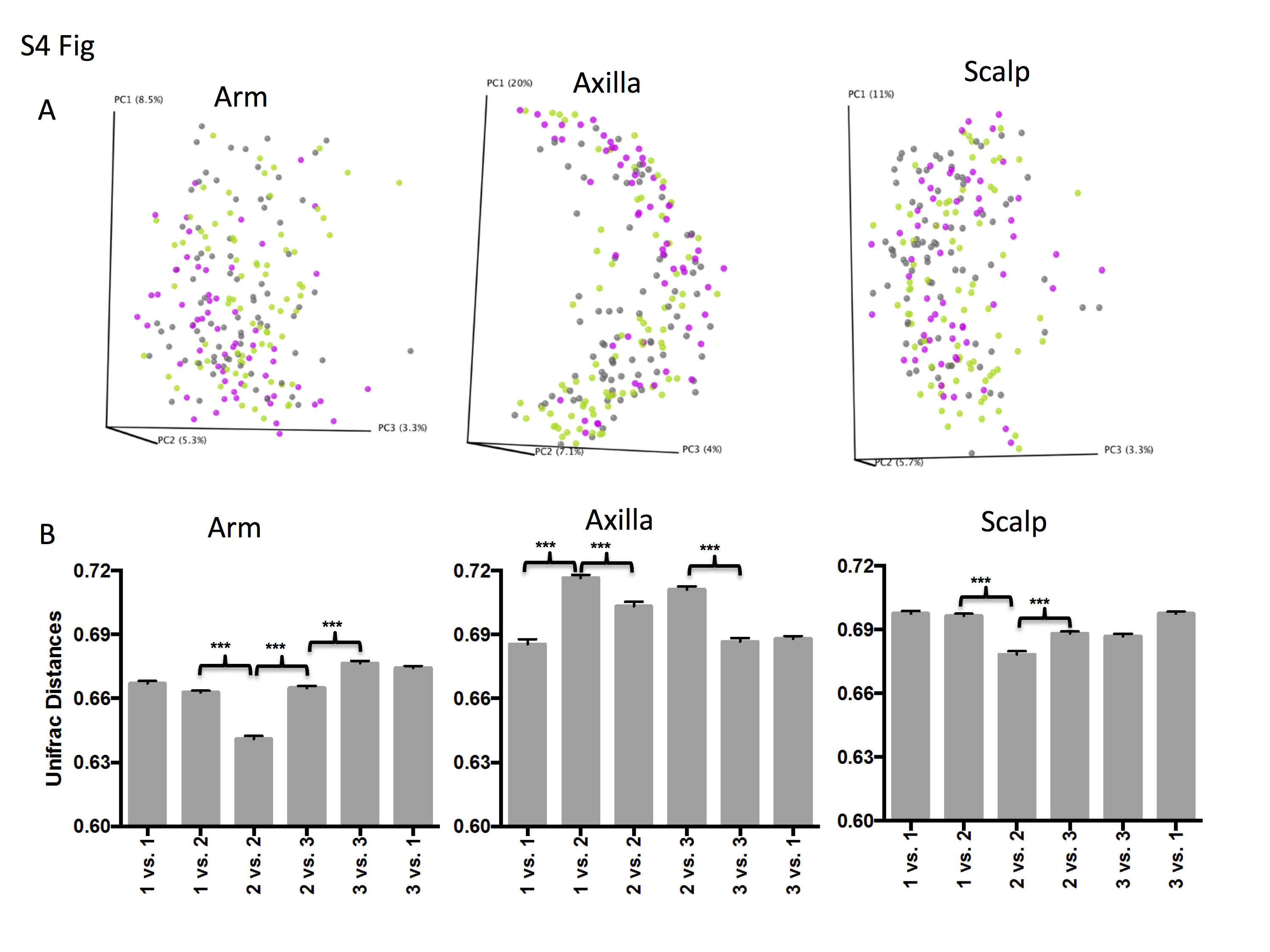

Supplement: S4 Fig — Panel A. Subjects were divided into three age strata [group 1:<25y, n = 77 (lime); group 2: 25–29y, n = 59 (pink); and group 3:>30y, n = 79 (gray)] and unweighted UniFrac analyses were done for each site sampled, and results visualized by PCoA. Panel B. Mean pairwise unweighted UniFrac comparing intra- and inter-group differences between groups 1, 2, and 3. ***p value <0.001. (TIFF) [file pone.0151990.s004.tiff]

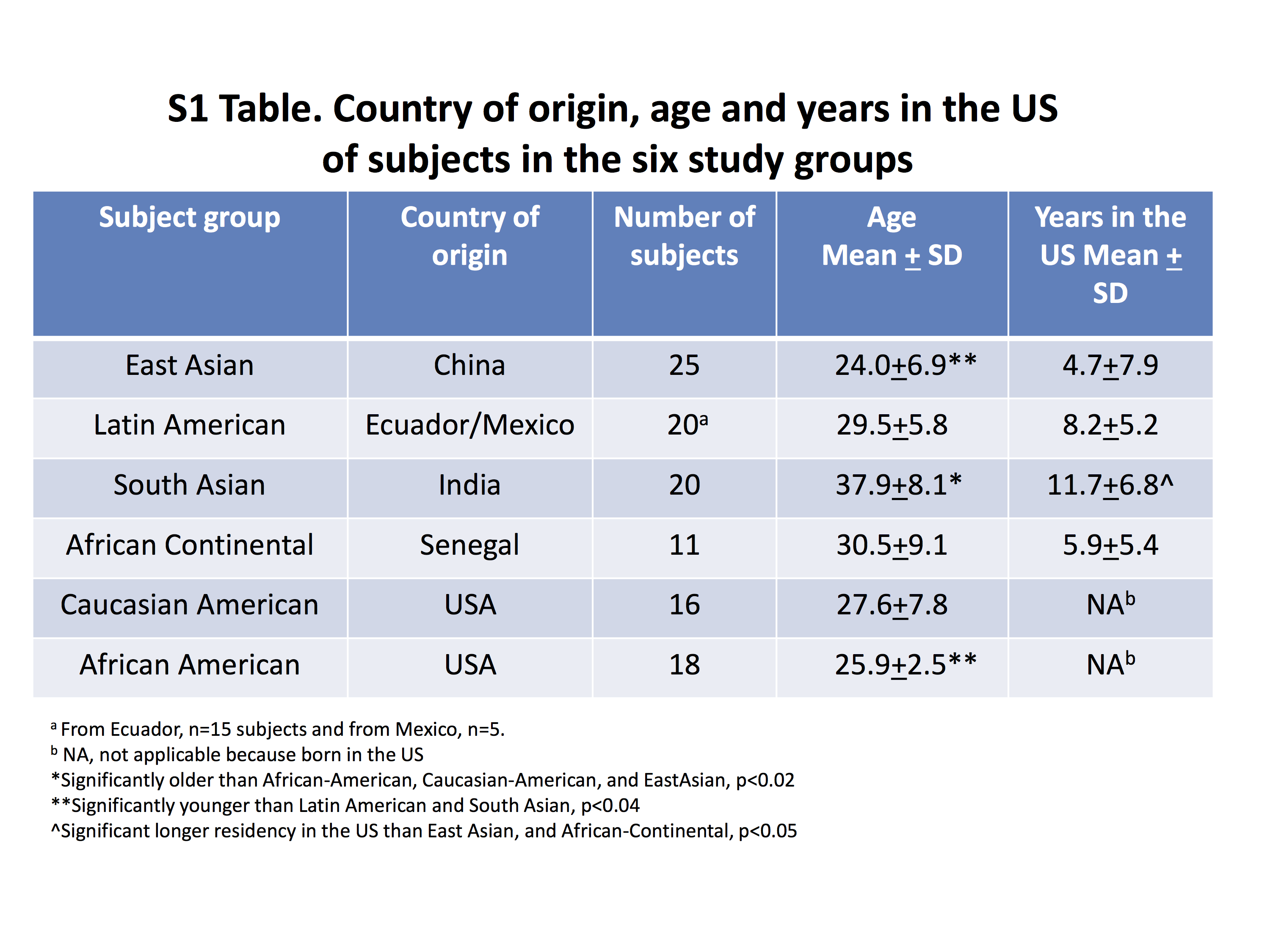

Supplement: S1 Table — a From Ecuador, n = 15 subjects and from Mexico, n = 5. b NA, not applicable because born in the US. *Significantly older than African-American, Caucasian-American, and EastAsian, p<0.02. **Significantly younger than Latin American and South Asian, p<0.04. ^Significant longer residency in the US than East Asian, and African-Continental, p<0.05. (TIFF) [file pone.0151990.s005.tiff]
